# Frequency and Prognostic Impact of Consistently Low Edmonton Symptom Assessment System Score in the Patients Treated with Palliative Radiotherapy

**DOI:** 10.7759/cureus.2032

**Published:** 2018-01-06

**Authors:** Carsten Nieder, Thomas A Kämpe

**Affiliations:** 1 Dept. of Oncology and Palliative Medicine, Nordland Hospital Trust

**Keywords:** palliative radiotherapy, edmonton symptom assessment system, prognosis, cancer, patient-reported symptoms

## Abstract

Introduction

Our department's standard work-flow includes assessment of all the patients with the Edmonton Symptom Assessment System (ESAS), a one-sheet questionnaire addressing 11 major symptoms and wellbeing on a numeric scale of zero-10, before the palliative radiotherapy (PRT). Based on previous research, we hypothesized that the patients with minimal or moderate total symptom burden might have better overall survival after the PRT than those with at least one higher symptom score.

Methods

We performed a retrospective analysis of 94 patients and calculated actuarial survival from the first day of the PRT (Kaplan-Meier method). We identified the patients with the score zero for all ESAS items (no symptoms), at least one item with score one-two (minimal symptoms), and at least one item with the score three (moderate symptoms).

Results

High proportions of the patients had ESAS scores zero- two for nausea (80%), sadness/depression (65%) and constipation (64%). The mean values were often in the range of two-four. Only one patient reported scores of zero throughout the questionnaire. He was treated for hematuria, a symptom that is not part of the ESAS. Three patients reported scores of zero-two throughout the questionnaire. Except for the performance status zero-one, their baseline characteristics were heterogeneous. Two patients reported scores not exceeding three for all items. These patients had excellent performance status, too. None of the six patients (6%) with relatively low ESAS scores of zero-three received care by the hospital's multidisciplinary palliative team. Only one was using opioid analgesics. The median survival for this small subset of six patients was six months, identical to the result for all the patients with higher symptom burden (p = 0.62).

Conclusion

The proportion of the patients with ESAS scores zero-three throughout the questionnaire was 6%, which resulted in the limited statistical power for the survival comparisons. The survival outcomes were similar. Before PRT, 94% of the patients reported at least one ESAS item of severity four-10. The symptoms not included in the questionnaire, e.g., hematuria might result in erroneous assignment to the low-symptom-burden group and obscure the prognostic impact of low ESAS symptom burden.

## Introduction

The Edmonton Symptom Assessment System (ESAS) is a short, one-sheet questionnaire addressing the well-being and the major symptoms, e.g., pain and nausea, on a numeric scale of zero-10 [1]. It has been employed in different clinical settings, including the registration of the patient-reported symptoms before the palliative radiotherapy [2-4]. The main purpose is to improve the clinical care, e.g., by adjusting medications and referring the patients to palliative care specialists, psycho-oncologists and other experts who might be able to enhance the quality of life [5-6]. Several aspects of the clinical research have also been addressed with this tool [7-8]. For example, the data suggest that ESAS scores provide prognostic information, mainly because severe symptoms are associated with shorter survival [9-10]. We hypothesized that the patients with minimal or moderate total symptom burden might have better overall survival after the palliative radiotherapy than those with at least one higher symptom score. In line with previous studies [11-12], we identified the patients with scores zero for all items (no symptoms), at least one item with score one-two (minimal symptoms), and at least one item with the score three (moderate symptoms). This study was performed to expand our ongoing efforts to develop prognostic models that support the decision making for personalized palliative approaches [13-15].

## Materials and methods

We performed a retrospective analysis of 94 patients who started the palliative radiotherapy during the time period between 2013-2015 and included the patients with the complete and incomplete treatment. The radiotherapy typically consisted of daily 3 Gray (Gy) or 4 Gy fractions or a single dose of 8 Gy fractions. The ESAS questionnaire was administered as part of our standard workup by a registered oncology nurse, before physician consultation and imaging for the treatment planning approximately one week before the radiotherapy. The statistical analysis was performed with the Statistical Package for the Social Sciences (SPSS) version 24 (IBM Corp., Armonk, New York). The actuarial survival was calculated from the first day of the radiotherapy (Kaplan-Meier method). Fifteen patients were still alive with a median follow-up of 18 months. The date of death was entered in the remaining 79 patients. The survival curves were compared by the log-rank test.

## Results

Most patients were male, elderly and had prostate or lung cancer with distant metastasis. The bone metastasis was common treatment indication, however, 11% of the patients had the non-metastatic disease and were treated for hematuria, dyspnea, and other local symptoms. Table 1 shows additional baseline characteristics.

**Table 1:** The baseline characteristics before the palliative radiotherapy. ECOG: Eastern Cooperative Oncology Group, RT: Radiotherapy, MPCT: multidisciplinary palliative care team. Some patients were treated with more than one target.

Variable	No	%
ECOG performance status		
0-1	36	38
2	30	32
≥ 3	28	30
Gender		
Male	67	71
Female	27	29
Primary tumor site		
Prostate	28	30
Breast	12	13
Lung (small cell)	1	1
Lung (non-small cell)	22	23
Colorectal	5	5
Bladder	5	5
Malignant melanoma	4	4
Kidney	4	4
Others	13	14
RT target types^1^		
Bone metastasis	60	64
Brain metastasis	10	11
Lymph node metastasis	4	4
Lung or thoracic wall	14	15
Prostate or bladder	9	10
Others	6	6
Patients without metastatic disease	10	11
Systemic cancer treatment		
No	19	20
Before RT	75	80
Opioid analgesics		
No	36	38
Yes	58	62
Steroids		
No	39	42
Yes	55	59
Care by MPCT	33	35
Median age, range, years	70 (49-91)	

As shown in Table 2, high proportions of the patients had ESAS scores zero-two (no or minimal symptoms), for nausea (80%), sadness/depression (65%) and constipation (64%). The mean values were often in the range of two-four.

**Table 2:** The Edmonton Symptom Assessment System (ESAS) before the palliative radiotherapy. ESAS zero on a scale from zero-10 with no symptoms.

Item	Mean	Range	% 0-2
Pain (not moving)	3	0-9	50
Pain (while moving)	4	0-10	34
Fatigue	4	0-10	39
Nausea	1	0-8	80
Dyspnea	3	0-10	55
Dry mouth	3	0-10	50
Appetite	4	0-10	45
Constipation	2	0-10	64
Anxiety/restlessness	3	0-10	56
Sleep	3	0-10	53
Sadness/depression	2	0-10	65
Overall wellbeing	4	0-10	37

Only one patient reported scores of zero throughout the questionnaire. He was treated for hematuria, a symptom that is not part of the ESAS. Three patients reported scores of zero-two throughout the questionnaire. Except for the performance status zero-one, their baseline characteristics were heterogeneous, as shown in Table 3.

**Table 3:** The overview of the six patients who had Edmonton Symptom Assessment System (ESAS) scores PS: performance status, NSCLC: non-small cell lung cancer, CTx: chemotherapy.

Patient nr.	Gender, age, PS	Tumor type, metastases	Target volume, dose	Chemotherapy, analgesics, steroids
1 (all items 0)	Male, 83, PS 2	Bladder cancer, lung/bone met.	Bladder (3 Gy x10)	No CTx, no opioid analgesics, no steroids
2 (max. 2)	Female, 67, PS 0	Breast cancer, 4 organs	Whole brain (2.5 Gy x15)	CTx, no opioids, steroids
3 (max. 2)	Male, 74, PS 1	Colon cancer, 4 organs	Bone (3 Gy x10)	CTx, opioids, no steroids
4 (max. 2)	Male, 75, PS 0	Rectal cancer, 3 organs	Bone (3 Gy x10)	CTx, no opioids, no steroids
5 (max. 3)	Female, 60, PS 0	NSCLC, brain only met.	Whole brain (2.5 Gy x15)	CTx, no opioids, no steroids
6 (max. 3)	Male, 70, PS 0	Mesothelioma, not met.	Thoracic wall (3 Gy x12)	CTx, no opioids, steroids

**Figure 1:**
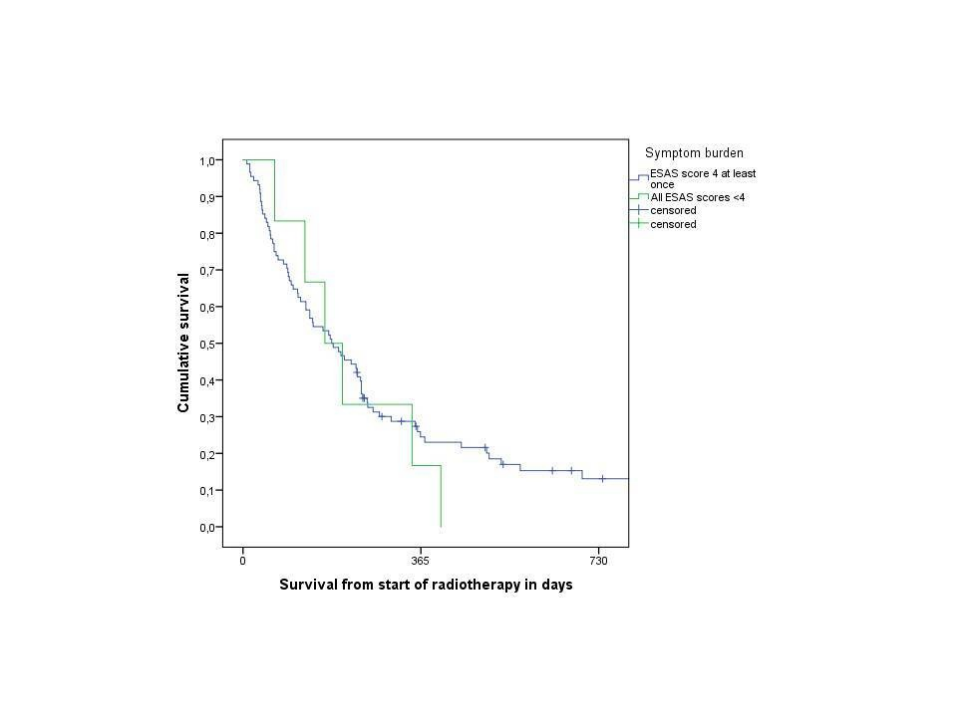
The actuarial overall survival after the radiotherapy.

Two patients reported scores not exceeding three for all items. These patients had excellent performance status too. None of the six patients (6%) with relatively low ESAS scores received care by the hospital's multidisciplinary palliative team. Only one was using opioid analgesics. The median survival for this small subset of six patients was six months, identical to the result for all the patients with higher symptom burden (Figure 1, p = 0.62).

## Discussion

The present study was performed as an extension of a previous one [16] and examined the impact of low ESAS symptom-burden of survival after the palliative radiotherapy. Previously, we performed standard uni- and multivariate- analyze where each ESAS item was dichotomized by the median. The multivariate model showed that appetite and pain were associated with survival, in addition to the performance status, administration of the systemic treatment and other variables. Other studies have also suggested that certain ESAS items influenced survival outcomes [9-10]. However, the results were not identical. Currently, these patient-reported symptoms are not included in commonly employed prognostic models, e.g., for brain metastasis and lung cancer [13-15, 17-18].

Other methodological approaches than dichotomization by median exist too. We hypothesized that the patients with minimal or moderate total symptom burden might have better overall survival than those with at least one higher symptom score. Therefore, we identified the patients with the score zero for all items (no symptoms), at least one item with score one-two (minimal symptoms), and at least one item with the score three (moderate symptoms). Surprisingly, few patients (6%) fell into these categories. Therefore, our survival analysis had limited statistical power. The six patients with low symptom burden were not among the group of long-term survivors. The median survival was similar in the two subsets of the patients with different ESAS scores. It was interesting to note that the patients with low scores were a heterogeneous group, however, most of them had a performance status zero-one and received the systemic therapy, rather than the radiation alone. As one might expect, these six patients were not managed by our multidisciplinary palliative team and only one of them used opioid analgesics. In other words, the low symptom burden was also reflected in the general patterns of care, and the different data were consistent.

The ESAS information may be used to triage the patients with a relatively severe symptom burden to different palliative measures. However, this tool is less comprehensive than the quality of life questionnaires [19-20] and lacks potentially relevant domains, such as hematuria, which was present in one of our patients with otherwise negligible symptoms. Future studies that aim at identification of the patients with favorable prognosis should, therefore, focus on other predictors, e.g., performance status, primary cancer type and extent of metastasis. Regarding the patient-reported symptoms, the methodology used in the present study appears less promising than employing median scores or scores ≥ 4, i.e., previously suggested approaches [9, 16].

## Conclusions

The proportion of the patients with ESAS scores zero-three throughout the questionnaire was 6%, which resulted in the limited statistical power for the survival comparisons. The survival outcomes were similar. Before PRT, 94% of the patients reported at least one ESAS item of severity four-10. The symptoms not included in the questionnaire, e.g., hematuria, might result in the erroneous assignment to the low-symptom-burden group and obscure the prognostic impact of the low ESAS symptom burden.
